# The cap-proximal RNA secondary structure inhibits preinitiation complex formation on *HAC1* mRNA

**DOI:** 10.1016/j.jbc.2022.101648

**Published:** 2022-01-28

**Authors:** Jagadeesh Kumar Uppala, Leena Sathe, Abhijit Chakraborty, Sankhajit Bhattacharjee, Anthony Thomas Pulvino, Madhusudan Dey

**Affiliations:** 1 Department of Biological Sciences, University of Wisconsin-Milwaukee, Milwaukee, Wisconsin, USA; 2 Center for Autoimmunity and Inflammation, Center for Cancer Immunotherapy, La Jolla Institute for Immunology, La Jolla, California, USA

**Keywords:** Ire1, Hac1, ER, unfolded protein response, eukaryotic translation initiation, cDNA, complementary DNA, eIF, eukaryotic translation initiation factor, ER, endoplasmic reticulum, Met-tRNA_i_^Met^, initiator methionyl-tRNA, MFE, minimum free energy, PIC, preinitiation complex, SC, synthetic complete, UI, 5′-UTR–intron, uORF, upstream ORF, WB, Western blot, WCE, whole cell extract, YEPD, yeast extract peptone dextrose

## Abstract

Translation of *HAC1* mRNA in the budding yeast *Saccharomyces cerevisiae* is derepressed when RNase Ire1 removes its intron *via* nonconventional cytosolic splicing in response to accumulation of unfolded proteins inside the endoplasmic reticulum. The spliced *HAC1* mRNA is translated into a transcription factor that changes the cellular gene expression patterns to increase the protein folding capacity of cells. Previously, we showed that a segment of the intronic sequence interacts with the 5′-UTR of the unspliced mRNA, resulting in repression of *HAC1* translation at the initiation stage. However, the exact mechanism of translational derepression is not clear. Here, we show that at least 11-base-pairing interactions between the 5′-UTR and intron (UI) are sufficient to repress *HAC1* translation. We also show that overexpression of the helicase eukaryotic initiation factor 4A derepressed translation of an unspliced *HAC1* mRNA containing only 11-bp interactions between the 5′-UTR and intronic sequences. In addition, our genetic screen identifies that single mutations in the UI interaction site could derepress translation of the unspliced *HAC1* mRNA. Furthermore, we show that the addition of 24 RNA bases between the mRNA 5′-cap and the UI interaction site derepressed translation of the unspliced *HAC1* mRNA. Together, our data provide a mechanistic explanation for why the cap-proximal UI–RNA duplex inhibits the recruitment of translating ribosomes to *HAC1* mRNA, thus keeping mRNA translationally repressed.

In all living organisms, mRNA carries the information that directs protein synthesis involving ribosomes, tRNA, and other factors in a process called translation (1, 2). Translation is a tightly coordinated and regulated process that ensures that an optimal amount of protein is produced, and the production of a specific protein is increased or decreased as needed. There are two major categories of translational regulation: global and transcript specific. In global regulation, the overall rate of translation is increased or decreased by inhibiting or activating one or more general translational components. In transcript-specific regulation, the translation of a given mRNA is controlled by the *cis* element(s) present in its UTRs and/or by the transacting regulatory protein(s) or noncoding RNA(s) (3). The *cis* elements include the upstream ORFs (uORFs), specific nucleotides flanking AUG start codon and the structured mRNA elements. As for example, four short uORFs control the *GCN4* mRNA translation in the budding yeast *Saccharomyces cerevisiae* (4), and two uORFs control the activating transcription factor *4* mRNA in humans (5). Like uORFs, an RNA secondary structure located within the 5′-UTR controls the *HAC1* mRNA translation in *S*. *cerevisiae* (6), whereas a tertiary-folded RNA structure, called the internal ribosomal entry site, controls the *cMYC* mRNA translation in humans (7).

The *S. cerevisiae HAC1* mRNA exists in a translationally repressed form in the cytoplasm by a unique mechanism involving its unspliced intron that interacts with the 5′-UTR to form an RNA duplex (Fig. 1*A*) (6). Previously, we and others have shown that the 5′-UTR–intron (UI)–RNA duplex inhibits the initiation of *HAC1* translation (6, 8). Under stress conditions, such as when unfolded proteins overaccumulate inside the endoplasmic reticulum (ER), *HAC1* mRNA colocalizes with an ER-resident RNase Ire1 that cleaves out the inhibitory intron (9, 10). tRNA ligase then ligates two cleaved exons (11), thus generating a matured mRNA that yields Hac1 protein (12, 13). Hac1 is a basic leucine-zipper transcription factor that binds to unfolded protein response element present in promoters of many ER-resident enzyme and chaperone genes (14), resulting in activation of their transcriptional program. Ultimately, the protein folding capacity of the cell is enhanced by this altered transcriptional program, which is collectively known as the unfolded protein response (15).

**Figure 1:**
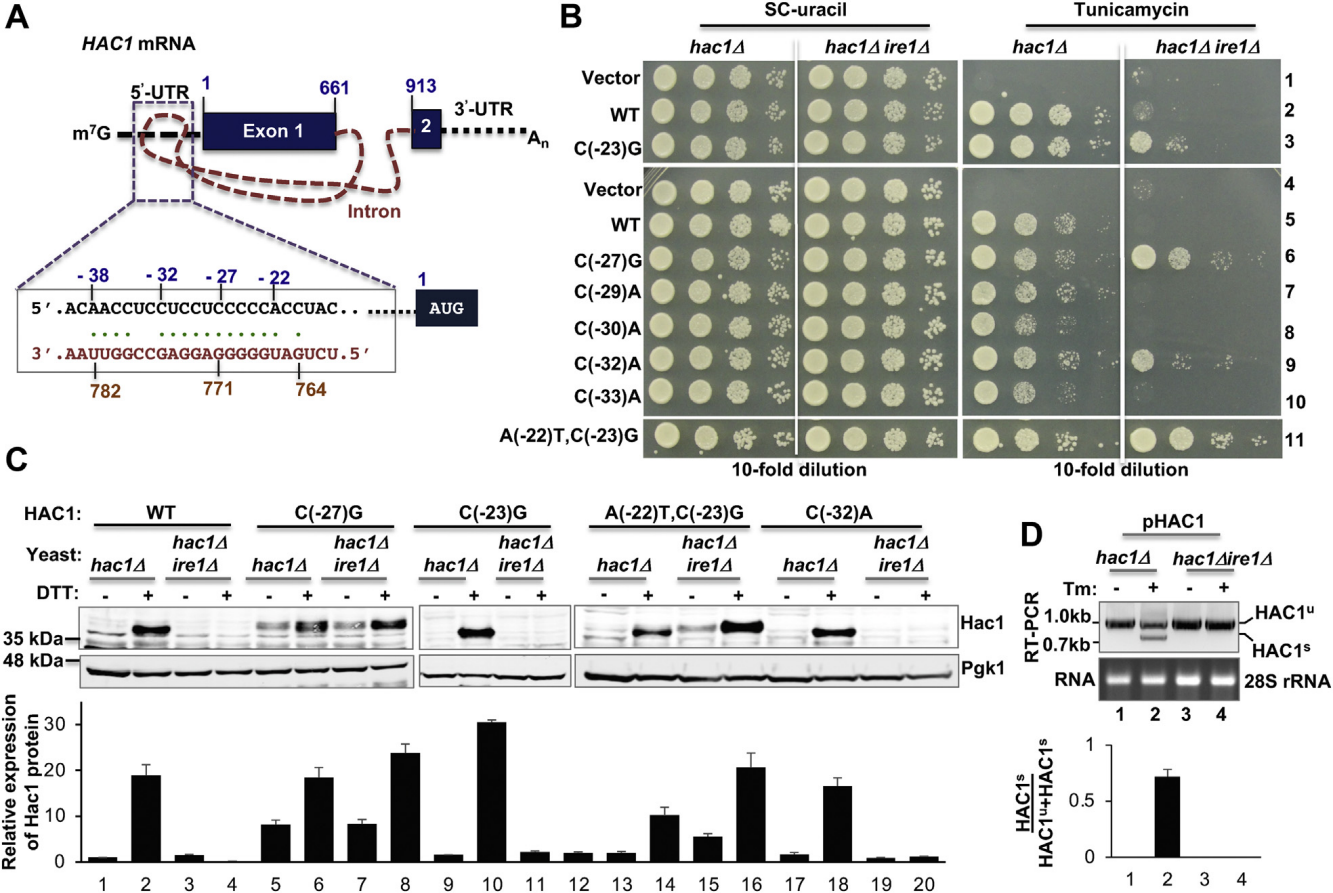
**Translational derepression of the unspliced *HAC1* mRNA by single or double base changes in the 5′-UTR•intron interaction site.***A*, schematic representation of *HAC1* mRNA. The (m^7^G) cap, 5′-UTR, and 3′-UTR (*black dotted lines*), two exons (*black boxes*), intron (*orange dashed line*), and polyadenylated tail (An) of *HAC1* mRNA are shown. The nucleotide composition of the 5′-UTR–intron interaction is shown. The nucleotide numbers are shown at the *top*. *B*, mutations at the 5′-UTR bases C(-23), C(-27), and C(-32) by adenine or guanine allow *hac1*Δ*ire1Δ* cells to grow under an endoplasmic reticulum (ER) stress condition. The *hac1Δ* and *hac1Δ ire1Δ* strains containing WT *HAC1* or its indicated mutant in *URA3* plasmids were grown overnight, serially diluted (considering 0.5 absorbance at 600 nm = 1) and spotted on the synthetic complete (SC) medium without uracil (SC-uracil) and on the same medium containing tunicamycin (0.2 μg/ml). *C*, the unspliced *HAC1* mRNA with specific 5′-UTR mutation can translate Hac1 protein. The *hac1Δ* or *hac1Δire1Δ* strain expressing indicated WT or mutant *HAC1* mRNA was grown in the presence (+) and absence (−) of DTT. Whole cell extracts were prepared and subjected to Western blot analysis using Hac1 and phosphoglycerate kinase 1 (Pgk1) antibodies. The Hac1 and Pgk1 protein band intensities were measured using ImageJ software. Each experiment was repeated at least twice, and the average ratios of those band intensities are shown below in the bar graph. *D*, ER stress condition activates *HAC1* mRNA splicing. The *hac1Δ* and *hac1Δire1Δ* strains expressing the WT *HAC1* from a plasmid (pHAC1) were grown in the presence (+) and absence (−) of tunicamycin. Total RNA was isolated (28S rRNA) and subjected to RT–PCR analysis to monitor the spliced (HAC1^s^) and unspliced (HAC1^u^) mRNA populations. The HAC1^s^ and HAC1^u^ RNA band intensities were measured. The ratios of HAC1^s^ and HAC1^s^ plus HAC1^u^ band intensities are shown in the bar graph.

The intron in *HAC1* mRNA functions as a *cis*-acting modulator of its mRNA's translation (Fig. 1*A*). Removal of this intron by the RNase Ire1 derepresses its translational initiation (6, 8). However, the exact mechanism of translational derepression at the initiation stage is not yet clear. Typically, several eukaryotic translation initiation factors (eIFs), ribosomes, and the initiator methionyl-tRNA (Met-tRNA_i_^Met^) work in concert to ensure that Met-tRNA_i_^Met^ finds the correct cognate start codon to initiate translation (16). Initiation of translation, according to the classical view, begins at the m^7^G-cap of mRNA with the formation of a ribonucleoprotein complex known as the preinitiation complex (PIC). PIC then travels along the 5′-UTR in search of a start codon in a process known as the ribosomal scanning. However, under certain conditions, cap-dependent translation initiation occurs without ribosomal scanning (17). Therefore, it is not yet clear how and to what extent the UI–RNA duplex in *HAC1* mRNA affects the assembly of ribosomes and other factors to form the PIC or its movement along the 5′-UTR.

In the present study, we reanalyzed both transcriptome and translatome data published for the budding yeast *S*. *cerevisiae* (18). In addition, we did the *in silico* analysis of RNA–RNA interaction between the 5′-UTR and intronic sequences. Along with these computational analyses, we provide *in vivo* evidence that the UI–RNA duplex sterically blocks the ribosomal access to the 5′-UTR near the m^7^G-cap of *HAC1* mRNA to form the PIC, thus keeping mRNA translationally repressed.

## Results

### Single base changes in the 5′-UTR derepress translation of the unspliced *HAC1* mRNA

Ruegsegger *et al.* (6) reported that a long-range base-pair interaction between sequences of the 5′-UTR (nucleotide positions from -38 to -20) and the intron (nucleotide positions from 764 to 782) keeps *HAC1* mRNA translationally repressed (Fig. 1*A*). We show that a single base-pair interaction between the RNA base C(-27) at the 5′-UTR and the G771 at the intronic region is important to keep the *HAC1* mRNA translationally repressed (8). Translational repression is released when the ER-resident endonuclease Ire1 cleaves the intron at positions G661 and G913 (19). Still, the detailed mechanisms underlying the translational repression and derepression of *HAC1* mRNA are not clear. In an effort to understand the mechanisms, we screened for single mutation(s), which would activate translation from the unspliced *HAC1* mRNA. For the mutational screen, we transformed an *Escherichia coli XL1-Red* mutator strain with a plasmid bearing a hemagglutinin-tagged *HAC1* gene and purified the mutated plasmid pool. The mutated plasmid pool was then introduced in the splice-deficient *hac1Δire1Δ* strain and screened for yeast colonies that were resistant to an ER stress–inducing agent tunicamycin. From the tunicamycin-resistant colonies, individual plasmids were rescued, retested, and sequenced to identify mutations. Apart from the C(-27)G mutation that we reported earlier (8), two single mutations (*i.e.*, C(-23)G and C(-32)A) were identified within the UI interaction site (Fig. 1*A*), which conferred resistance to tunicamycin (Fig. 1*B*, rows 3 and 9).

The aforementioned results suggested that 11 RNA bases at the 5′-UTR ranging from A(-22) to C(-32) likely form a core 11-bp RNA duplex with the intronic bases from U766 to G776, which tunes the *HAC1* mRNA translation (Fig. 1*A*). Thus, we extended our studies and mutated almost all base-pairing nucleotides of the UI–RNA duplex and determined their relative contribution in translational repression. Nucleotides C(-29), C(-30), and C(-33) were individually mutated to adenine, thus generating three plasmids bearing C(-29)A, C(-30)A, and C(-33)A mutation at the 5′-UTR of *HAC1* mRNA. In addition, we mutated the adenine at position -22 of the *HAC1*-C(-23)G derivative to thymine (T), thus generating a *HAC1*-A(-22)T, C(-23)G double mutant. The mutant plasmids were then individually introduced in both *hac1Δ* and *hac1Δire1Δ* strains. The resulting strains were tested for their ability to survive under a condition of ER stress stimulated by tunicamycin.

The *hac1Δ* strain containing a vector plasmid or the same plasmid bearing a WT *HAC1* allele grew on synthetic complete (SC) medium (Fig. 1*B*, SC-uracil, rows 1, 2, 4, and 5). However, the *hac1Δ* strain grew on the SC medium containing tunicamycin only when expressed a WT *HAC1* allele (Fig. 1*B*, tunicamycin, rows 2 and 5). These results are consistent with the previous findings that *HAC1* gene provides an essential function to alleviate ER stress. Like WT *HAC1*, each *HAC1-5*′-*UTR* mutant [*HAC1*-C(-23)G, *HAC1*-C(-27)G, *HAC1*-C(-29)A, *HAC1*-C(-30)A, *HAC1*-C(-32)A, *HAC1*-C(-33)A, or *HAC1*-A(-22)T, C(-23)G] allowed the *hac1Δ* cells to grow on the tunicamycin medium (Fig. 1*B*). Western blot (WB) analysis showed that Hac1 protein was produced when cells were grown in the presence of an ER stressor DTT (Fig. 1*C*, lanes 2, 6, 10, 14, and 18) or tunicamycin (data not shown). These data collectively suggest that the 5′-UTR mutations have little or no effect on Hac1 protein expression induced by ER stress.

Unlike *hac1Δ* strain, the *hac1Δire1Δ* strain containing a WT *HAC1* was unable to grow on the tunicamycin medium (Fig. 1*B*, rows 2 and 5). As expected, Hac1 protein was not detected in those cells grown in the presence of tunicamycin (data not shown) or DTT (Fig. 1*C*, lane 4), suggesting that the tunicamycin-sensitive phenotype was due to lack of Hac1 protein expression. Furthermore, we tested whether the loss of protein expression was due to the loss of *HAC1* mRNA splicing. Both *hac1Δ* and *hac1Δire1Δ* strains containing a WT *HAC1* allele on the *URA3* plasmid were grown in the presence of DTT. Total RNA was isolated from the DTT-stressed cells and subjected to RT–PCR analysis to examine the spliced (HAC1^s^) and unspliced (HAC1^u^) populations of mRNA. RT–PCR analysis showed only unspliced mRNA species in the *hac1Δire1Δ* strain expressing a WT *HAC1* allele (Fig. 1*D*, lanes 3 and 4). Both *HAC1*^*s*^ and *HAC1*^*u*^ mRNA species were observed in the *hac1Δ* strain grown in the presence of an ER stressor DTT (Fig. 1*D*, lane 2). These results are consistent with other studies that demonstrate that Ire1 function is required for *HAC1* mRNA splicing. The spliced mRNA yields Hac1 protein that induces the ER stress response.

The *hac1Δire1Δ* strain containing the plasmid-borne *HAC1* or its 5′-UTR mutant [*i.e.*, *HAC1*-C(-29)A, *HAC1*-C(-30)A, and *HAC1*-C(-33)A] was unable to grow on the tunicamycin medium (Fig. 1*B*, rows 7, 8, and 10). In contrast, the same *hac1Δire1Δ* strain containing the *HAC1*-C(-23)G, *HAC1*-C(-27)G, *HAC1*-C(-32)A, or *HAC1*-A(-22)T, C(-23)G mutant was able to grow on the tunicamycin medium (Fig. 1*B*, rows 3, 6, 9, and 11). Notably, cells containing the *HAC1*-A(-22)T, C(-23)G, or *HAC1*-C(-27)G mutant grew more rapidly than cells containing the *HAC1*-C(-23)G or *HAC1*-C(-32)A mutant (Fig. 1*B*). The results suggested that Hac1 protein was likely produced from the unspliced mRNA starting at the AUG codon of exon 1 until the stop codon UGA at nucleotide 690 of the adjacent intron. This translational product of unspliced *HAC1* mRNA is known as “Hac1^u^” protein (20). Hac1^u^ protein, like the Hac1 protein translated from the spliced mRNA (*i.e.*, Hac1^s^ protein), is a transcription factor with a basic leucine-zipper domain followed by a transcription activation domain, differing in only 10 residues of its C terminus (20). However, Hac1^u^ protein is short lived, differently modified, and less active transcription factor (19).

To determine that Hac1^u^ protein was produced from the aforementioned 5′-UTR mutants in the *ire1Δ hac1*Δ strain, whole cell extracts (WCEs) were prepared from the respective strains grown in the SC medium and subjected to WB analysis. A significant amount of Hac1 protein was produced (∼25% compared with WT) in the *hac1Δire1Δ* strain containing the *HAC1*-(C-27)G (Fig. 1*C*, lanes 5–8) and *HAC1*-A(-22)T, C(-23)G mutants (Fig. 1*C*, lanes 13–16). Consistent with our recent report (21), we also observed that Hac1^u^ expression was induced almost twofold when cells were grown in the presence of the ER stressor DTT (Fig. 1*C*, lanes 5–8). However, Hac1^u^ protein was almost undetectable in the *hac1Δire1Δ* strain containing the *HAC1*-(C-23)G or *HAC1*-(C-32)A mutant, even when cells were grown in the presence of DTT (Fig. 1*C*, lanes 11, 12, 19, and 20). It appears that a low amount of Hac1^u^ protein was sufficient to confer a mild tunicamycin-resistant phenotype (Fig. 1*B*, rows 3 and 9). A possibility that we cannot rule out that the low amount of Hac1^u^ protein produced from the unspliced *HAC1*-(C-23)G or *HAC1*-C(-32)A mRNA was likely degraded rapidly mediated by a degrader protein, Duh1, as previously reported (22). Taken together, our data suggest that single mutations at the 5′-UTR bases C(-23), C(-27), and C(-32) derepressed translation of the unspliced *HAC1* mRNA, whereas mutations at the bases C(-29), C(-30), and C(-33) did not have any noticeable effect. Together, these results suggest that RNA bases C(-23), C(-27), and C(-32) play major regulatory roles in translational repression of *HAC1* mRNA.

**Figure 2:**
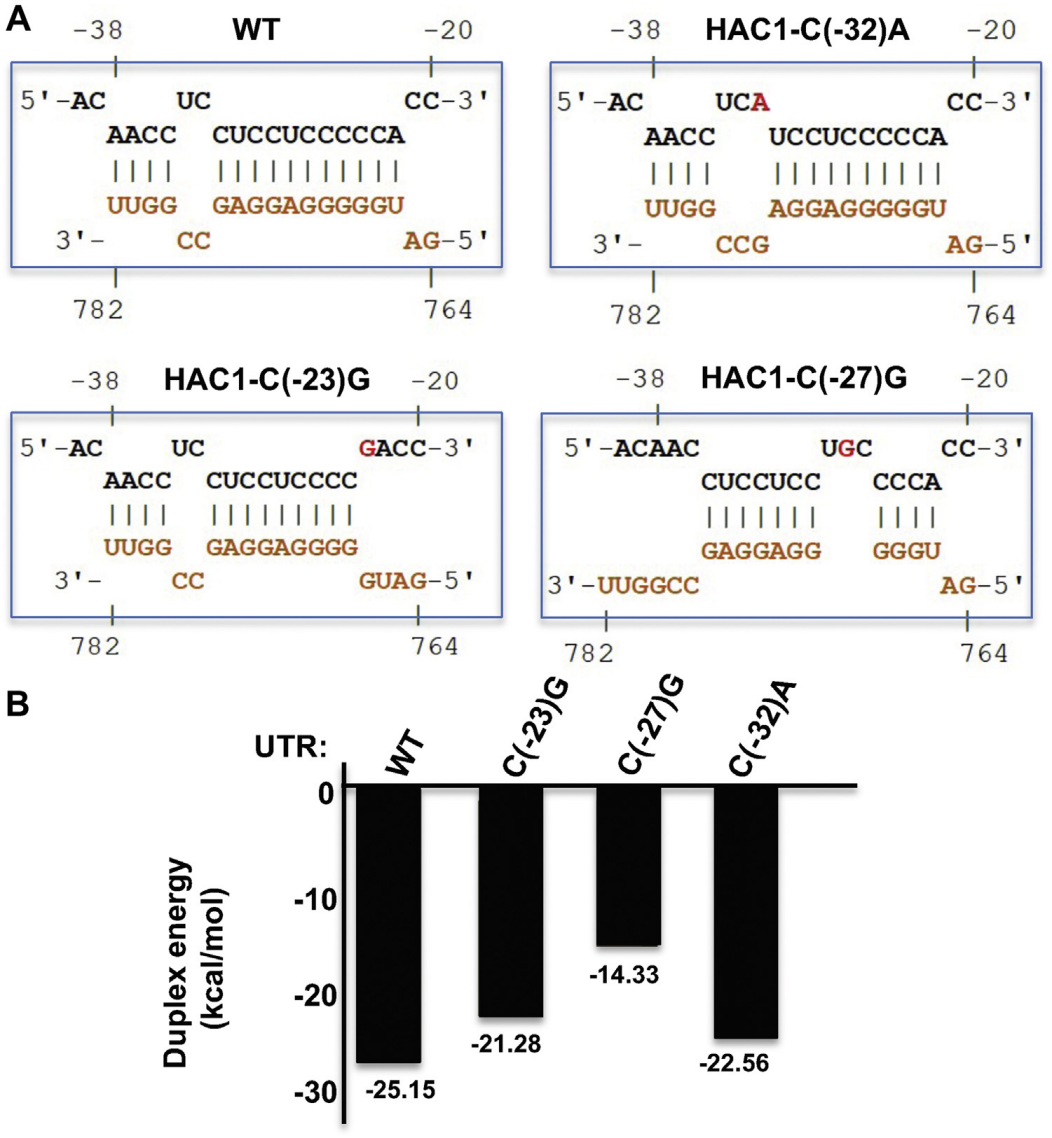
**Quantitative analyses of base-pair interaction between sequences of the 5′-UTR and the intron.***A*, predicted base-pairing interactions between sequences of the intron and the 5′-UTR or its mutants. The RNA–RNA hybrid interactions are predicted using the IntaRNA software (23). *B*, mutations at the 5′-UTR bases lower the duplex interaction energy. The bar diagram shows duplex energies of 5′-UTR–intron interactions.

Next, we used the IntaRNA program (23) to predict the RNA–RNA hybrid formation between sequenced of the 5′-UTR and the intron. Nineteen RNA bases at the 5′-UTR (the base position from -20 to -38) were predicted to form 15-bp interactions with the 20 intronic bases (the base position from 764 to 782) with a duplex binding energy (E) of −25.15 kcal/mol (Fig. 2, *A* and *B*). The low E value of the duplex energy indicates a thermodynamically stable hybrid, capable of forming a strong RNA secondary structure. About 13 bps were predicted when the 5′-UTR base C(-23) and C(-32) were mutated to guanine and adenine, respectively (Fig. 2). The respective E values were −21.28 kcal/mol mutant and −22.56 kcal/mol (Fig. 2). Interestingly, we found that only 11 bps were predicted when the RNA base C(-27) was mutated to guanine, and the E value was of −14.33 kcal/mol (Fig. 2). These higher E values upon mutations at RNA bases C(-23), C(-27), and C(-32) suggested that each mutation weakened the strength of base-pair interaction. Indeed, the relatively higher E values upon the C(-27)G mutation likely explains why Hac1^u^ expression was more in cells harboring the *HAC1*-C(-27)G mutant than in cells harboring the *HAC1*-C(-23)G or *HAC1*-(C-32)A mutant (Fig. 1*B*). Concurrently, these computational data strongly corroborate with the phenotypic observations that cells containing the *HAC1*-C(-27)G mutant were more resistant to tunicamycin than cells containing the *HAC1*-C(-23)G or *HAC1*-C(-32)A mutant (Fig. 1*B*, compare rows 3, 6, and 9).

Taken together, these molecular, genetics, and computational data suggest that single mutations at the 5′-UTR [*e.g.*, C(-23)G, C(-27)G, and C(-32)A] can partially disrupt the long-range base-pairing interaction with the intron, resulting in leaky expression of Hac1^u^ protein. Indeed, these results lead us to speculate that ribosome was able to produce Hac1^u^ protein from these 5′-UTR mutants with a weak base-pair interaction, more likely utilizing an alternate mechanism for cap-dependent translation initiation.

### Eleven base-pairing interactions between the sequence of the 5′-UTR and the intron are sufficient to repress *HAC1* mRNA translation

**Figure 3:**
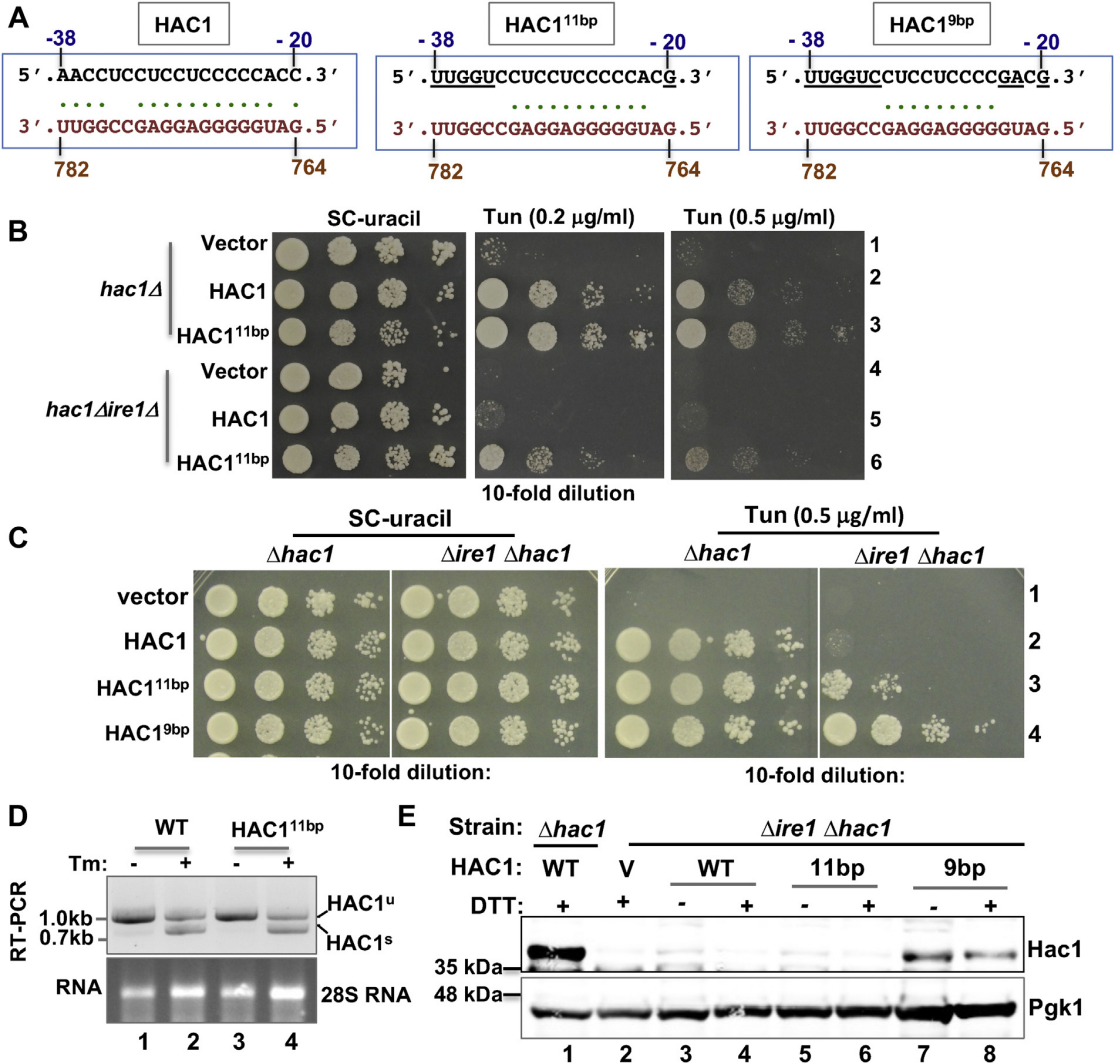
**About 11-bp interactions between sequence of the 5′-UTR and the intron are sufficient to repress *HAC1* mRNA translation.***A*, the base-pair interaction between the sequences of the 5′-UTR (*black*) and intron (*orange*) in WT and mutant *HAC1* mRNAs. Base-pair interactions are shown by *dots*. The nucleotide numbers are shown at the *top* and *bottom*. *B* and *C*, analysis of yeast growth. The *hac1Δ* or *hac1Δire1Δ* strains expressing the WT and *HAC1*^9bp^ or *HAC1*^11bp^ mutant were tested for growth on the synthetic complete (SC) medium and the same SC medium containing the indicated concentration of tunicamycin (Tm). *D*, Tm-induced endoplasmic reticulum (ER) stress activates splicing of *HAC1*^11bp^ mRNA. The *hac1Δ* strain expressing WT or mutant HAC1^11bp^ was grown in the presence (+) and absence (−) of Tm. Total RNA was isolated (28S rRNA is shown) and subjected to RT–PCR analysis to monitor the spliced (HAC1^s^) and unspliced (HAC1^u^) mRNA populations. Experiment was repeated twice, and average ratios are shown. *E*, expression of Hac1 protein from the *HAC1*^9bp^ mutant without ER stress. The *hac1Δ* and *hac1Δire1Δ* strains expressing WT HAC1 and *HAC1*^9bp^ or *HAC1*^11bp^ mutant were grown in the presence (+) and absence (−) of DTT. Whole cells extracts were prepared and subjected to Western blot analysis using anti-Hac1 and anti-Pgk1 antibodies. pgk1, phosphoglycerate kinase 1.

Ruegsegger *et al.* (6) show that the interaction between 16 RNA bases of the 5′-UTR and the intron leads to repression of *HAC1* mRNA translation. Consistent with this report, the IntaRNA analysis predicts 15 possible base-pair interactions (Fig. 3*A*). Here, we experimentally determined the minimum number of base-pair interactions required for the translational repression in *HAC1* mRNA. First, we engineered the *HAC1* gene by mutating five DNA bases coding for five 5′-UTR RNA bases as follows. Four RNA bases at positions -35 to -38 (AACC) were replaced by RNA base UUGG, and cytosine at the position -20 was replaced by a guanine (Fig. 3*A*). This derivative, herein referred to as *HAC1*^*11bp*^, was expected to preserve only a stretch of 11 continuous UI base-pair interactions (Fig. 3*A*). Second, we further engineered the *HAC1*^*11bp*^ allele by mutating two more DNA bases coding for the RNA bases at positions -22 and -23, thus generating a mutant that was expected to preserve only 9 bp interactions, herein referred to as *HAC1*^*9bp*^ derivative (Fig. 3*A*). We expressed individually the *HAC1*^*11bp*^ and *HAC1*^*9bp*^ mutants in both *hac1Δ* and *hac1Δire1Δ* strains and examined how these mutations affected the cell's ability to adapt to and overcome ER stress.

The *hac1Δ* strain expressing the *HAC1*^*11bp*^ mutant grew on the SC medium and the same medium containing a low (0.2 μg/ml) or high (0.5 μg/ml) concentration of tunicamycin (Fig. 3*B*, row 3 and Fig. 3*C*, row 3). RT–PCR analysis showed both unspliced and spliced mRNA in cells expressing *HAC1*^*11bp*^ mutant, like in cells expressing a WT allele, when they were grown in the presence of DTT (Fig. 3*D*). These data suggest that the *HAC1*^*11bp*^ mutant neither affect the fitness of cells to grow under an ER stress condition nor impair *HAC1* mRNA splicing. Interestingly, we observed that the *hac1Δire1Δ* strain harboring the *HAC1*^*11bp*^ mutant grew slowly on the SC medium containing tunicamycin at a low concentration (0.2 μg/ml, Fig. 3*B*). Growth was almost abolished at a high concentration of tunicamycin (0.5 μg/ml, Fig. 3*B*). WB showed that Hac1 protein was almost undetectable in cells harboring *HAC1*^*11bp*^ mutant even when they were grown in the presence of DTT (Fig. 3*E*, WB, lanes 5 and 6). These results suggested that mutations weakened the strength of interaction between the 5′-UTR and intronic sequences, resulting in leaky translation of Hac1 protein and partial survival of cells under a mild ER stress condition. These results further suggested that a low amount of Hac1 protein was sufficient to confer the tunicamycin-resistant phenotype and that Hac1 protein produced from the unspliced mRNA was likely degraded rapidly as reported earlier (22). In contrast, the *hac1Δire1Δ* strain harboring the *HAC1*^*9bp*^ mutant grew normally on the SC medium containing tunicamycin at concentration of 0.5 μg/ml (Fig. 3*C*, row 4). Growth was correlated with the expression of Hac1 protein (Fig. 3*E*, WB, lanes 7 and 8). These results suggested that mutations at both positions -22 and -23 of *HAC1*^*11bp*^ allele eliminated the base-pair interaction between the UI, or 9 bp interactions between the UI were not sufficient to repress translation. Nevertheless, these results suggest that minimally the regions predicted to have 11 base-pairing interactions are sufficient and near threshold for inhibition of *HAC1* mRNA translation.

### An RNA hairpin of 11-bp stem inhibits translation of the matured *HAC1* mRNA

The RNAfold software (24) predicts that the minimum free energy (MFE) of the 11-bp RNA hairpin in *HAC1* mRNA is approximately −22 kcal/mol. It has been shown previously that an RNA hairpin (MFE = −30 kcal/mol) was capable of inhibiting *in vitro* translation when positioned at 12 nucleotides away from the 5′-cap but not when positioned 52 nucleotides away (25). In contrast, an iron-responsive element with higher free energy (MFE = −5.86 kcal/mol) combines with iron regulatory proteins and inhibits translation initiation when positioned at <60 nucleotides away from the 5′-cap (26). Thus, we sought to determine how and to what extent, a similar 11 base-paired RNA hairpin modulated the general protein synthesis under physiological conditions. Specifically, we monitored translation from an intron-less *HAC1* mRNA by adding an 11 base-paired RNA hairpin near the 5′-cap.

**Figure 4:**
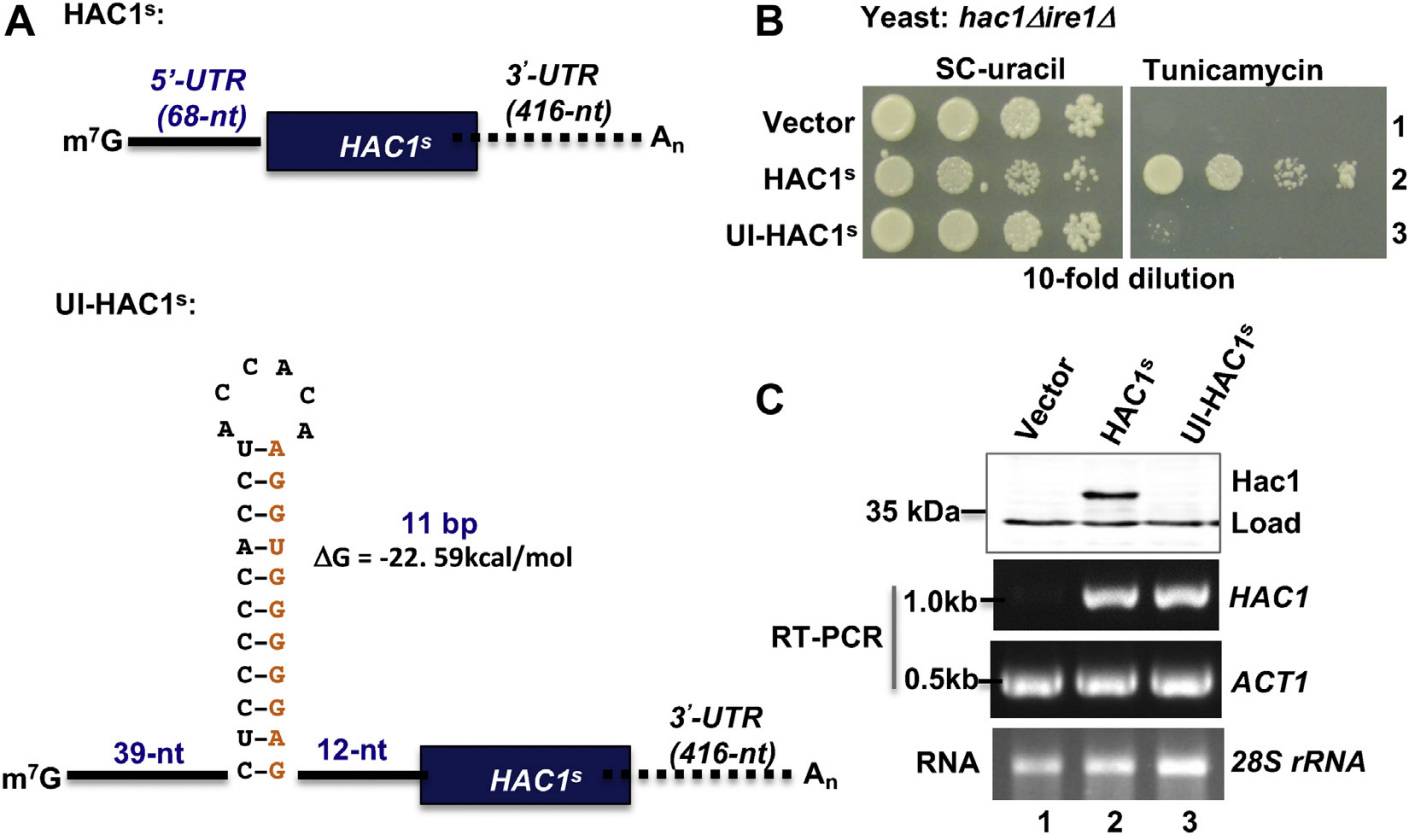
**An 11-bp RNA hairpin at the 5′-UTR inhibits translation of intron-less *HAC1* mRNA.***A*, schematic representation of intron-less *HAC1* mRNA (HAC1^s^) and its derivative. The (m^7^G) cap, 5′-UTR (*black solid line*), ORF (*black box*), 3′-UTR (*black dotted lines*), and the polyadenylated tail (An) of *HAC1* mRNA are shown. A sequence of 11 intron nucleotides (*orange color*) was added at the 5′-UTR in reverse orientation, which is supposed to make an 11-bp RNA hairpin at the position 39 nucleotides away from the 5′-cap. *B*, analysis of yeast growth. The *hac1Δire1Δ* strain expressing the intron-less *HAC1* mRNA (HAC1^s^) or its derivative UI-HAC1^s^ was tested for growth on synthetic complete (SC) and tunicamycin media at 30 °C for 2 days. *C*, analysis of both mRNA and protein expressions from UI-HAC1^s^ construct. *Upper panel*, whole cell extracts were prepared from an *ire1Δ hac1*Δ strain harboring the UI-HAC1^s^ construct and subjected to Western blot analysis using anti-Hac1 antibodies. *Lower panel*, total RNA was isolated (28S rRNA) from the strains shown in *B* and was subjected to RT–PCR analysis to monitor the expressions of *HAC1* and *ACT1* mRNAs using the respective gene-specific primers.

To this end, we made two Hac1 constructs: HAC1^s^ and UI-HAC1^s^. In HAC1^s^ construct, the entire intron sequence was deleted from the D63 plasmid bearing the *HAC1* gene under its natural promoter and terminator (see *Experimental procedures* section). The intron-less HAC1^s^ construct was expected to constitutively express a *HAC1* mRNA with a 5′-UTR (68 bases), a protein-coding region (717 bases), and a 3′-UTR (416 bases) (Fig. 4*A*) (18). In the UI-HAC1^s^ construct, 11 complementary nucleotides of the *HAC1-5*′-*UTR* sequence (-32 to -22) were inserted at position -11 of the 5′-UTR of the HAC1^s^ construct. The UI-HAC1^s^ construct was expected to produce an mRNA with an extra 11-bp 5′-UTR sequence, including an 11-bp RNA hairpin positioned ∼39 bases away from the 5′-cap (Fig. 4*A*).

The *hac1Δire1Δ* strain harboring the HAC1^s^ construct was able to grow on both SC and tunicamycin media (Fig. 4*B*) because of constitutive expression of Hac1 protein (Fig. 4*C*). The slow growth of the cells on the SC medium (Fig. 4*B*) suggested that the constitutive expression of Hac1 protein reduced the cell growth. Unlike the HAC1^s^ construct, the chimeric UI-HAC1^s^ construct did not allow the *hac1Δire1Δ* strain to grow on the tunicamycin medium (Fig. 4*B*). Similar levels of *HAC1* mRNA were observed in cells containing HAC1^s^ and chimeric UI-HAC1^s^ constructs (Fig. 4*C*); however, Hac1 protein expression was almost undetectable in cells containing the chimeric UI-HAC1^s^ construct (Fig. 4*C*). These data suggest that the UI-HAC1^s^ construct likely produces an mRNA containing a 5′-UTR sequence that might self-fold to form an 11-bp RNA hairpin near the 5′-cap, which is inhibitory to translation initiation. These results suggest that translation inhibition by the UI–RNA duplex is mediated by a unique mechanism; however, this is in line with the published report that the secondary structures (MFE ≤ −22 kcal/mol) decrease translational efficiency (27). Collectively, these results further support our observation that a stretch of 11-bp interactions in the UI–RNA duplex is sufficient to repress *HAC1* mRNA translation (Fig. 3).

### Translational derepression of unspliced *HAC1*^11b^ mRNA by helicase eIF 4A

Along with ribosomes, a set of initiation factors coordinately assemble on the 5′-cap to initiate translation (16). Therefore, it is possible that the UI interaction may prevent the assembly of one/two of these components. To test this possibility, we examined if *HAC1* mRNA translation could be tuned or boosted by high dose of any of these translational components. Using the high-copy-number plasmids, we overexpressed a few translational components, in particular, two key components of the scanning ribosome (*i.e.*, eIF1 and eIF1A), a subunit of ternary complex (*i.e.*, eIF2α), a subunit of the eIF3 complex (*i.e.*, eIF3a), subunits of the cap-binding complex eIF4F (*i.e.*, eIF4E and eIF4A), the GTPase-activating protein eIF5 and GTPase eIF5B (16). None of these translational factors was able to derepress translation of unspliced *HAC1* mRNA and promote growth of the *ire1Δ* strain on the tunicamycin medium (data not shown), suggesting that overexpression of each of these translational components was insufficient to derepress *HAC1* mRNA translation when the intron was not spliced.

**Figure 5:**
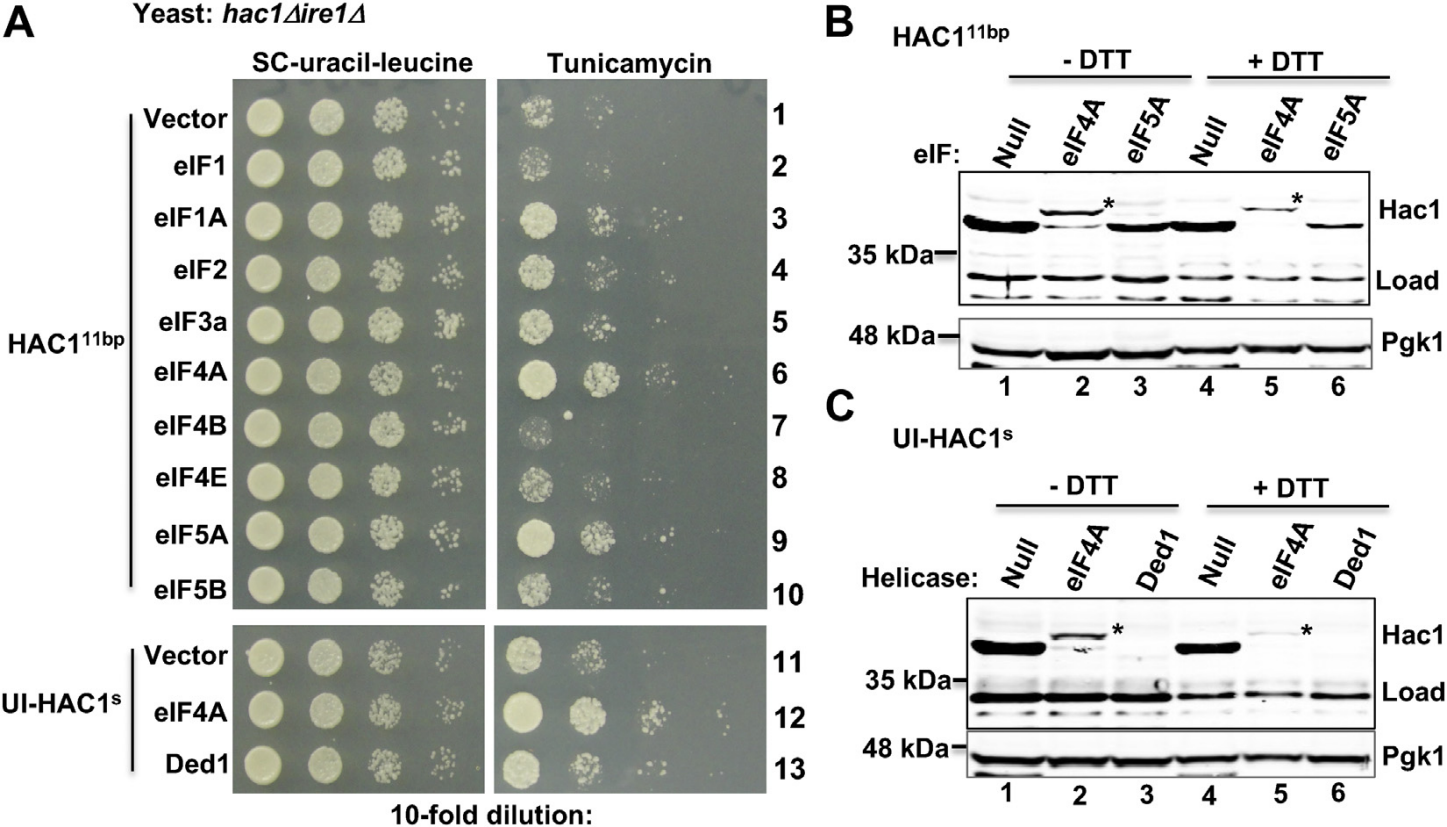
**Derepression of *HAC1***^***11bp***^**mRNA translation by the helicase eIF4A.***A*, analysis of yeast growth. The *hac1Δire1Δ* strain expressing the indicated *HAC1*^*11bp*^ or UI-HAC1^s^ mutant and the indicated eIFs or helicases from a high-copy-number plasmid were tested for growth on the synthetic complete (SC) and tunicamycin media at 30 °C for 2 days. *B* and *C*, analysis of Hac1 protein expression. Whole cell extracts from the *hac1Δire1Δ* strain coexpressing the indicated *HAC1*^*11bp*^ or UI-HAC1^s^ mutant and the respective eIFs or helicases were subjected to Western blot analysis using anti-Hac1 and anti-Pgk1 antibodies. The Hac1 protein bands are indicated with *asterisks*. Nonspecific bands are also shown as loading control. eIF4A, eukaryotic translation initiation factor 4A; Pgk1, phosphoglycerate kinase 1.

Then, we overexpressed the aforementioned translational components in the *hac1Δire1Δ* strain expressing the crippled *HAC1*^*11bp*^ mutant. Interestingly, we observed that overexpression of the helicase eIF4A enhanced cell growth on the tunicamycin medium (Fig. 5*A*, rows 6 and 9). Consistent with the increased tunicamycin-resistant phenotype, the Hac1 protein expression was detected in cells grown in the absence or the presence of DTT (Fig. 5*B*, lanes 2 and 5). However, Hac1 expression was almost undetected when eIF5A was overexpressed (Fig. 5*B*, lanes 3 and 6). It was surprising that a major prominent band below the Hac1 protein consistently appeared on the WB of cell lysates obtained from the *ire1Δhac1Δ* strain (Fig. 5*B*, lanes 1, 3, 4, or 6). Specifically, we observed a prominent WB band of whole cell lysate prepared from the *ire1Δhac1Δ* strain harboring a *URA3* plasmid carrying the *HAC1* gene and a high-copy *LEU2* plasmid (Fig. 5*B*, lane 1 and Fig. 5*C*, lane 1). However, such prominent band was not observed on the WB of cell lysate prepared from the *ire1Δhac1Δ* strain harboring only the *URA3*-based plasmid carrying the *HAC1*^*11b*^ allele (Fig. 3*E*, lanes 5 and 6). Currently, we do not have any specific explanation but can speculate that unspliced *HAC1* mRNA is constitutively be translated from an alternate start site in the presence of the high-copy *LEU2* plasmid. Nonetheless, these results suggest that overexpression of eIF4A was able to unwind the crippled RNA secondary structure in the *HAC1*^*11bp*^ mutant, and Hac1 protein was produced from the unspliced mRNA (Fig. 5*C*).

The DEAD-box helicase eIF4A and a related helicase Ded1 are known to melt the 5′-UTR secondary structure and significantly contribute to PIC formation, ribosomal scanning, and start codon selection (28, 29). Therefore, to further determine if these helicases eIF4A and Ded1 could overcome the inhibitory effect of RNA secondary structure (Fig. 5*A*) and promote translation from the *UI-HAC1*^s^ mRNA, we overexpressed them from a high-copy-number plasmid in *hac1Δire1Δ* cells containing the *UI-HAC1*^s^ construct. Interestingly, we observed that the helicase eIF4A, but not Ded1, enhanced the tunicamycin-resistant phenotype (Fig. 5*A*, row 12). Consistent with the increased growth on the tunicamycin medium, we observed expression of Hac1 protein in those cells grown in the presence or the absence of DTT (Fig. 5*C*, lanes 2 and 5), suggesting that a high dose of eIF4A was able to overcome the inhibitory effect of the RNA secondary structure. These data suggest that eIF4A plays an important role for translational derepression of *HAC1* mRNA containing a secondary structure at 5′-UTR.

### Insufficient room for ribosome and helicase recruitment on the 5′-UTR of *HAC1* mRNA

**Figure 6:**
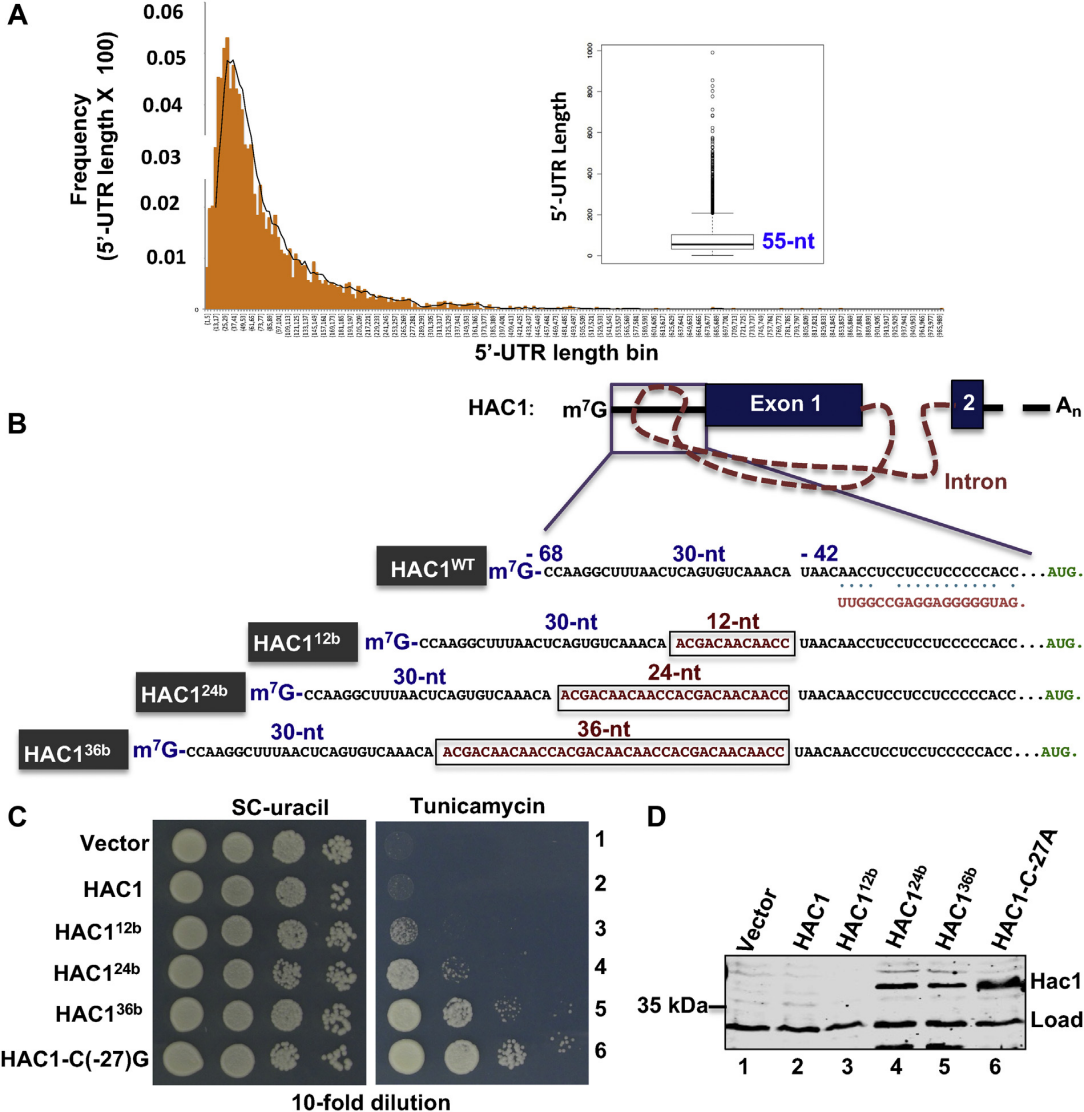
**Limited space between the 5′-cap and the 5′-UTR–intron site precludes the preinitiation complex formation.***A*, the median length of 5′-UTRs of *Saccharomyces cerevisiae* is 55 nucleotides. The transcriptome data were retrieved from the supporting information of an article published by Nagalakshmi *et al.* (18), and the frequencies of the respective 5′-UTR length are binned as shown in the diagram. The median length of 5′-UTRs is shown in the box plot. *B*, nucleotide compositions of the 5′-UTR sequence of WT *HAC1* and its derivatives. About 12, 24, or 36 RNA bases were inserted at the indicated site of the 5′-UTR. The intronic sequence that binds to the 5′-UTR is shown. *C*, analysis of yeast growth. A *hac1Δire1Δ* strain expressing the WT *HAC1* or its indicated derivatives (HAC1^12bp^, HAC1^24bp^, or HAC1^36bp)^ was tested for growth on synthetic complete (SC) and tunicamycin media. *D*, analysis of Hac1 protein expression. Whole cell extracts (WCEs) were prepared from the strains shown in (*C*) and subjected to Western blot analysis using the anti-Hac1 antibody.

We reanalyzed the budding yeast *S. cerevisiae* transcriptome data (18) and observed that 23% of mRNAs contain a 5′-UTR of ≤30 nucleotides and most mRNAs (48%) have a 5′-UTR of ≥55 nucleotides, and the median length of 5′-UTRs is of 55 nucleotides (Fig. 6*A*). Specifically, we found that the length of the *HAC1* 5′-UTR is 68 nucleotides long, and the UI–RNA duplex is positioned at about 30 RNA bases away from the 5′-cap (Fig. 6*B*). The RNAfold WebServer (24) software did not indicate that these 30 RNA bases adopt any specific secondary structure. Combining these analyses with the Ribo-Seq information that an individual ribosome typically covers ∼30 bases in an mRNA molecule (30, 31), we posit three possible scenarios for the RNA duplex–mediated translational repression of *HAC1* mRNA. First, a sequence of 30 RNA bases might not provide a sufficient landing space for the assembly of a PIC. Second, the UI–RNA duplex sterically blocks the assembly of PIC on the 5′-UTR. Third, the PIC may assemble on the 5′-UTR but be unable to unwind the nearby UI RNA duplex.

To test the aforementioned hypotheses, we increased the 5′-UTR length, particularly the length between the 5′-cap and UI–RNA duplex, under the assumption that the longer the space the more available room will exist for translation factors binding, resulting in less inhibition of translation by the nearby RNA duplex. Therefore, we added 12 bases (ACGACAACAACC, taken from position -7 to -18) at the position -43 of the 5′-UTR, thus increasing the 5′-UTR length by 12 bases and creating a new *HAC1* mutant referred to as *HAC1*^*12b*^ mutant (Fig. 6*B*). Similarly, we further increased the length by 24 and 36 RNA bases adding the extra 12 (ACGACAACAACC) and 24 bases (ACGACAACAACC ACGACAACAACC), thus generating *HAC1*^*24b*^ and *HAC1*^*36b*^ mutants, respectively (Fig. 6*B*). Then, we monitored the expression of Hac1 protein from these *HAC1* derivatives in the splice-deficient *ire1Δ hac1Δ* strain.

An *hac1Δire1Δ* strain containing an empty vector or the same vector containing a WT *HAC1* or its derivative *HAC1*^*12b*^ was unable to grow on the tunicamycin medium (Fig. 6*C*, rows 1, 2, and 3). Consistent with the tunicamycin-sensitive phenotype, Hac1 protein expression was almost undetectable (Fig. 6*C*, WB, lanes 1, 2, and 3). In contrast, the *ire1Δ hac1Δ* cells containing the *HAC1*^*24b*^ or *HAC1*^*36b*^ mutant were able to grow on the tunicamycin (Fig. 6*A*, rows 4 and 5), like the strain expressing the *HAC1*-C(-27)G mutant plasmid (Fig. 6*A*, row 6). Consistent with the tunicamycin-resistant phenotype, a significant amount of Hac1 protein was detected in these cells (Fig. 6*D*, WBs, lanes 4, 5, and 6). Given that the *HAC1*-C(-27)G mutant (Fig. 1*C*) allowed cells to produce Hac1 protein from the unspliced mRNA, we interpret that the longer 5′-UTR lengths in *HAC1*^*24b*^ and *HAC1*^*36b*^ mutants allow cells to translate Hac1 protein from the unspliced mRNA.

We further interpret that the PIC is likely able to form on the 5′-UTR near the cap, which starts scanning along the 5′-UTR in search for an initiation codon. The intrinsic RNA unwinding activity of the scanning ribosome (32) and eIF4A enables the unwinding of the UI–RNA duplex for translation. Of note, the 5′-UTR length of *HAC1*^*24b*^ mRNA before the UI RNA duplex is 54 nucleotides, similar to the predicted average length of 5′-UTR in the budding yeast *S. cerevisiae*. Thus, this study provides an example of a broader phenomenon that 5′-UTRs of ≥55 nucleotides in length in *S*. *cerevisiae* may be important for efficient translation of 48% mRNAs as mentioned earlier.

### Translational derepression of the unspliced *HAC1*-AUG^-42^,A1G^24b^ mRNA

**Figure 7:**
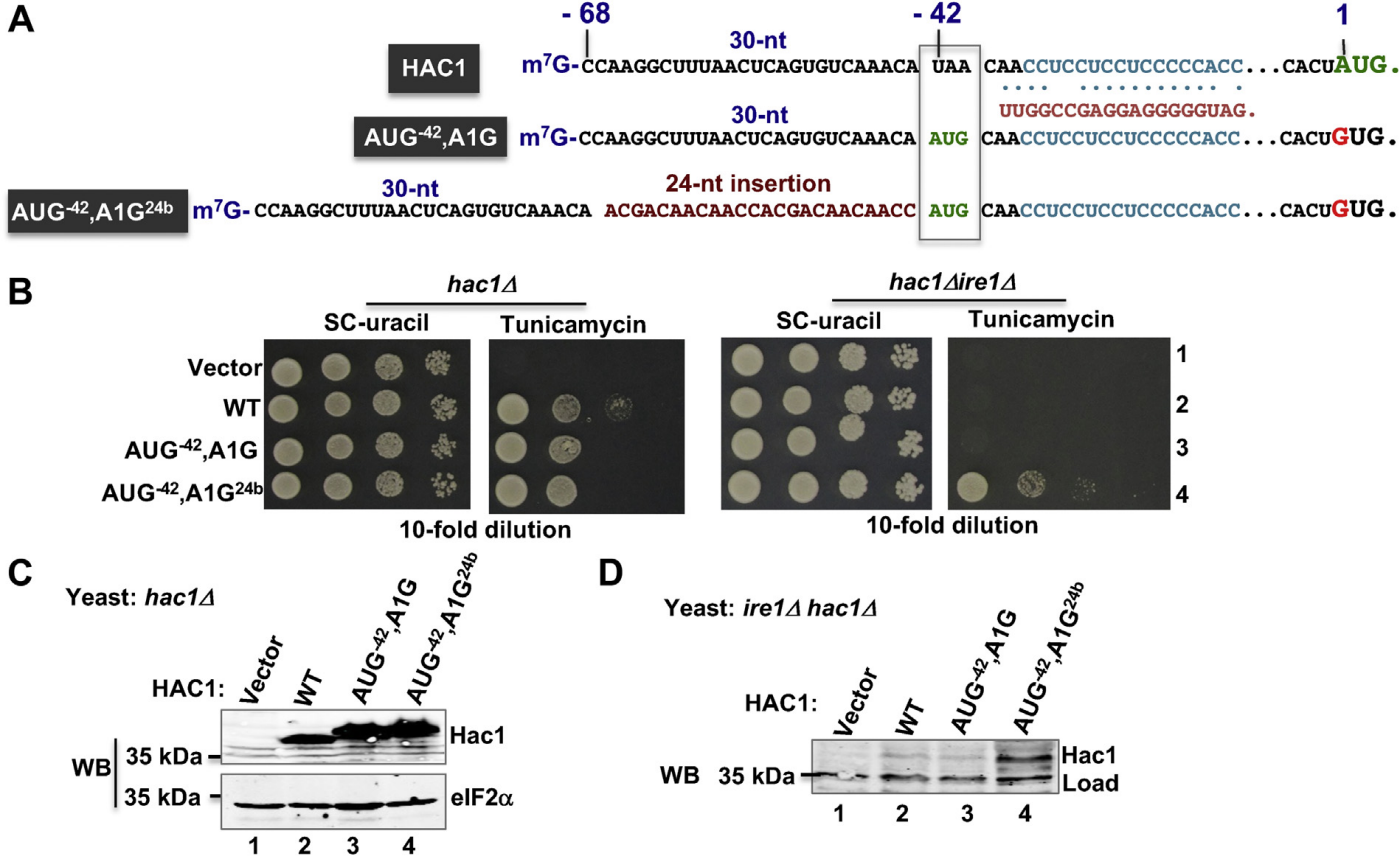
**Translational derepression in an engineered *HAC1-AUG***^***-42***^**,*A1G***^***24b***^**mRNA.***A*, the nucleotide compositions of the 5′-UTR sequences of the HAC1-AUG^-42^,A1G and *HAC1*-AUG^-42^,A1G^24b^ mRNAs. The scheme shows that a start codon AUG is inserted at position -42 and the authentic AUG codon is mutated to GUG. *B*, analysis of yeast growth. The *hac1Δ* and *hac1Δire1Δ* strains expressing the indicated WT *HAC1* and its derivatives were tested for growth on synthetic complete (SC) and tunicamycin media. *C* and *D*, analysis of Hac1 protein expression. Whole cell extracts from the *hac1Δ* and *hac1Δire1Δ* strains expressing the indicated WT *HAC1* and its derivatives were subjected to Western blot analysis using anti-Hac1 antibody. As a control, the same blot was probed with an eIF2α (eukaryotic translation initiation factor 2alpha) antibody.

Translation from the unspliced *HAC1* mRNA with an increased length of 5′-UTR suggests that 30 RNA bases between the 5′-cap and UI–RNA duplex did not provide sufficient space for the assembly of the PIC. We further confirm these results using a synthetic *HAC1-AUG*^*-42*^,*A1G* derivative, in which adenine of the normal AUG start codon was mutated to guanine and an in-frame AUG start codon was inserted by replacing the UAA RNA bases located at the 5′-UTR positions -42 to -40 (Fig. 7*A*). This *HAC1-AUG*^*-42*^,*A1G* mutant, like a WT *HAC1* allele, allowed the *hac1Δ* strain to grow on the tunicamycin medium (Fig. 7*B*). Consistent with the tunicamycin-resistant phenotype, Hac1 protein was detected in cells grown in the presence of DTT (Fig. 7*C*), suggesting that the synthetic *HAC1* mRNA behaved like a WT allele. It is to be noted that the migration of the Hac1 protein expressed from cells containing the *HAC1-AUG*^*-42*^,*A1G* mutant was slightly slower than the Hac1 protein expressed from cells containing the WT allele (Fig. 7*C*, WB, compare lanes 2 and 3). The slower migration was due to expression of Hac1 protein from the upstream AUG codon, which is larger (252 amino acids) than the WT protein (238 amino acids). Unlike the *hac1Δ* strain, the *hac1Δ ire1Δ* strain containing the *HAC1-AUG*^*-42*^,*A1G* mutant neither grew on the tunicamycin medium (Fig. 7*B*, *hac1Δ ire1Δ*, row 3) nor produced any Hac1 protein (Fig. 7*D*, WB, lane 3). These results suggest that either the PIC was unable to form on the 5′-UTR or the inserted AUG start codon in the 5′-UTR of unspliced mRNA remained inaccessible to the ribosomal complex because of the nearby RNA secondary structure.

To facilitate the accessibility of the AUG codon to the ribosomal complex, we added extra 24 RNA bases before the UI–RNA duplex of *HAC1-AUG*^*-42*^,*A1G* mutant, thus generating a *HAC1-AUG*^*-42*^,*A1G*^*24b*^ mutant (Fig. 7*A*). The *HAC1-AUG*^*-42*^,*A1G*^*24b*^ mutant allowed the *hac1Δ ire1Δ* strains to grow on the tunicamycin medium (Fig. 7*B*, row 4) and produced a detectable amount of Hac1 protein under normal condition (Fig. 7*D*, WB, lane 4). These data suggest that the extra 24 RNA bases before the AUG start codon allow ribosomes to form the PIC that likely starts traveling down the mRNA and finally recognizes the AUG start codon resulting in translation of the unspliced mRNA.

### The helicase eIF4A is associated with the translationally repressed *HAC1* mRNA

**Figure 8:**
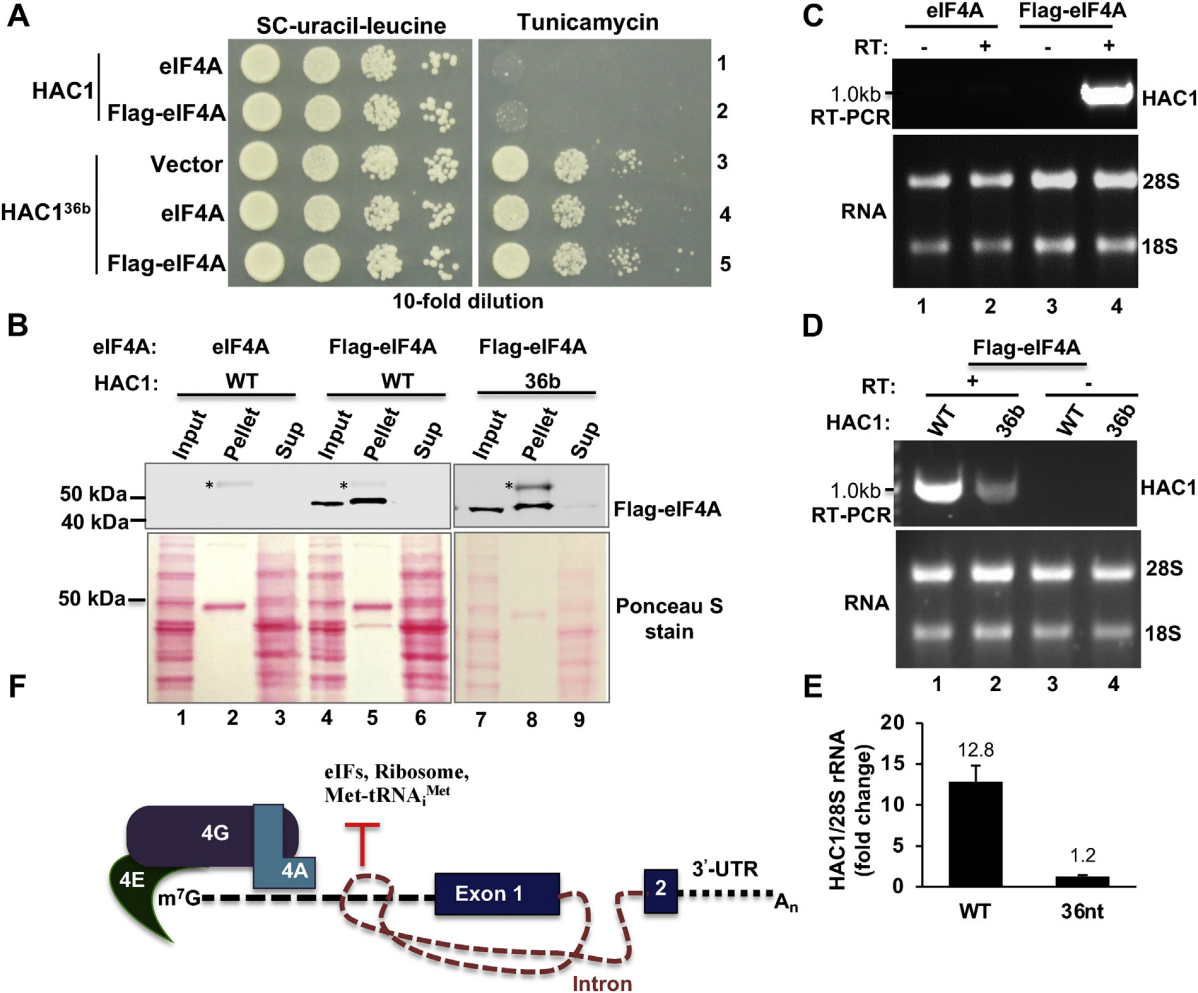
**Helicase eIF4A is associated with the translationally repressed *HAC1* mRNA.***A*, an *ire1Δhac1Δ* strain expressing the indicated WT HAC1 or its derivative HAC1^36b^ from a low-copy *URA3* plasmid and the indicated untagged or FLAG-tagged eIF4A from a low-copy *LEU2* plasmid were grown on synthetic complete (SC) and tunicamycin media without uracil and leucine. *B*, pulldown of the eIF4A–RNA complex. *Upper panel*, whole cell extracts (WCEs) were prepared from the *ire1Δhac1Δ* cells coexpressing the indicated eIF4A and *HAC1* mRNA. From the WCE, the FLAG-eIF4A protein was then pulled down by anti-FLAG agarose resin. Input (10%), pellet, and supernatant (sup) fractions were analyzed by Western blotting using an anti-FLAG antibody. *Lower panel*, the Ponceau S staining pattern of the lysates blotted on the nitrocellulose membrane. The nonspecific band in the pellet fractions is indicated by “∗” symbols. *C* and *D*, helicase eIF4A is predominantly associated with the translationally repressed *HAC1* mRNA. WCEs were prepared from the *ire1Δhac1Δ* cells expressing the indicated eIF4A and *HAC1* mRNA. From WCEs, the FLAG-eIF4A protein–RNA complex was pulled down (pellet fraction) by anti-FLAG agarose resin. Total RNA from the eIF4A–RNA complex was purified and subjected to RT–PCR analysis with *HAC1*-specific primers. As a control, the same reaction was carried out without RT (*upper panel*). *E*, quantification of the helicase eIF4A–*HAC1* mRNA association. Experiments in (*D*) were repeated thrice. Average intensity of the amplified *HAC1* complementary DNA from three experiments is shown with standard error. *F*, the proposed model depicting how an m^7^G cap-proximal RNA duplex (*black ladder*) formed by sequences of the 5′-UTR and intron inhibits translation by precluding the recruitment of eIFs and ribosome on the 5′-UTR of *HAC1* mRNA bound to the cap complex consisting of eIF4E, eIF4G, and eIF4A. eIF4A, eukaryotic translation initiation factor 4A.

We investigated whether the UI RNA duplex would sterically block the recruitment of helicase eIF4A to the 5′-UTR. Thus, we monitored the association of helicase eIF4A with the translationally rerepressed and derepressed *HAC1* mRNAs. WCEs were prepared from an *ire1Δhac1Δ* strain coexpressing untagged or FLAG-tagged eIF4A from a high-copy *LEU2* plasmid and translationally repressed *HAC1* or translationally derepressed *HAC1*^*36b*^ mRNA mutant from a low-copy *URA3* plasmid (see the *Experimental procedures* section). From the WCEs, the FLAG-eIF4A protein was then pulled down by anti-FLAG agarose resin. Both pellet and supernatant fractions were collected and analyzed by WB. As expected, the FLAG-eIF4A was predominantly pulled down with the pellet fraction (Fig. 8*B*, WB, lanes 5 and 8). Total RNA was also isolated from both pellet and supernatant fractions. The isolated RNA from the pellet fraction was then subjected to RT–PCR analysis using *HAC1*-specific primers. Amplification of *HAC1* complementary DNA (cDNA) was observed in the reaction mixture containing RT (Fig. 8*C*, lane 4) but not in the reaction without RT (Fig. 8*C*, lane 3), suggesting that amplification of *HAC1* was based on cDNA synthesis. Interestingly, we observed that amplification of *HAC1* in cells expressing the translationally repressed WT *HAC1* was ∼10-fold more than cells expressing the translationally derepressed *HAC1*^*36b*^ mRNA mutant (Fig. 8, *D* and *E*). These data suggest that the translationally repressed *HAC1* mRNA is associated with the helicase eIF4A, and majority of eIF4A dissociates from the translationally active mRNA. Typically, eIF4A is a component of the cap-binding complex eIF4F, containing other two major subunits: an eIF4E and a large scaffolding protein eIF4G (3). Thus, it appears that translationally derepressed *HAC1* mRNA is bound to cap-binding protein complex and exported from the nucleus.

## Discussion

In the present study, we show that three RNA bases (C-23, C-27, and C-32) at the UI interaction site play a major role in the translational regulation of *HAC1* mRNA. We also show that minimally 11 bp interactions between the UI are required to repress *HAC1* mRNA translation. Furthermore, we show that overexpression of the helicase eIF4A can derepress translation of an unspliced *HAC1* mRNA containing an 11-bp interaction between the 5′-UTR and intronic sequences. Finally, we demonstrate that, consistent with the possibility, 30 RNA bases before the UI interaction site precludes the recruitment of the translationally competent PIC on the *HAC1-5*′-*UTR*.

The rate-limiting step of translation is the initiation, which occurs typically by a cap-dependent or cap-independent manner (16). In cap-independent translation, an mRNA structure referred to as the “internal ribosomal entry site” directly binds a subset of initiation factors, the 40S ribosomal subunit and Met-tRNA_i_^Met^, resulting in the recognition of the start codon by Met-tRNA_i_^Met^. In contrast, the start codon recognition by Met-tRNA_i_^Met^ in the cap-dependent translation is thought to occur in three steps. First, a 43S-PIC (a complex of the 40S ribosomal subunit, initiation factors, and the initiator Met-tRNA_i_^Met^) assembles on the mRNA 5′-cap bound to an eIF4F protein complex, consisting of a cap-binding protein eIF4E, a scaffold eIF4G, and the helicase eIF4A (16, 33). Second, the 43S-PIC travels along the 5′-UTR in search of a start codon (16, 34). The ribosomal scanning is facilitated by the helicase eIF4A and/or Ded1, which unwind any secondary structure present in the 5′-UTR (28). Third, the Met-tRNA_i_^Met^ of the 43S-PIC recognizes the start codon, and all initiation factors dissociate from the 40S ribosome. Then, the 60S ribosomal subunit joins the 40S subunit to yield an 80S ribosomal complex (35), where the Met-tRNA_i_^Met^ is placed right over the start codon. The 80S ribosome then proceeds to decode mRNA into a protein molecule. Consistent with this linear model of ribosomal scanning, we observed that the addition of extra space between the 5′-cap and the UI–RNA duplex allowed cells to derepress translation of the unspliced *HAC1* mRNA (Fig. 6).

A growing body of evidence suggests that the length of the 5′-UTR and its contextual fold determine the translational efficiency of mRNAs (36). On the 5′-UTR next to the 5′-cap, the PIC assembles and then scans for the start codons along the 5′-UTR, except for some mRNAs with extremely short 5′-UTR (<12 bases), which undergo scanning-free initiation (37). In essence, both PIC assembly and scanning are major control points of translation initiation. 5′-UTRs vary in lengths, ranging from a few to thousands of nucleotides with a median length of ∼55 bases in the budding yeast *S*. *cerevisiae* and ∼218 bases in humans, *Homo sapiens* (36). 5′-UTRs may also self-fold into a simple hairpin or more complex secondary and tertiary structures that can block the ribosomes to form the PIC (36). These secondary structures inhibit the translational efficiency. In this study, we show that minimally 11-bp interactions between the 5′-UTR and intronic sequences are required to keep *HAC1* mRNA translationally repressed. Consistently, we show that addition of an 11-bp RNA hairpin inhibits Hac1 expression from an intron-less *HAC1* mRNA (Fig. 4). Then, we demonstrate that the inhibitory effect of this structured mRNA is overcome by increased dosage of eIF4A (Fig. 5). However, we observed that translationally repressed *HAC1* mRNA is associated with the helicase eIF4A (Fig. 8). Therefore, it appears that the UI RNA duplex likely interferes with the eIF4A activity.

In the budding yeast *S*. *cerevisiae*, high-throughput sequencing technologies including SHAPE-Seq (selective 2′-hydroxyl acylation analyzed by primer extension) (38) and Frag-Seq (fragmentation sequencing) reveal that >90% of 5′-UTRs could form secondary structures (39). In addition, chemical probing and structural analysis of *S*. *cerevisiae* mRNAs showed that the protein-coding regions are more structured than their leader or trailer sequences (39). This is likely because most RNAs in cells are covered by ribosomes, helicases, and/or RNA-binding proteins. Indeed, these approaches significantly advance our knowledge about the structural dynamics of cellular mRNAs; however, our knowledge is still limited on how self-folded RNA elements control localization, transport, and translational efficiency of mRNA. The regulatory effects of the predicted structured mRNA on translational efficiency require an intensive compensatory mutational analysis.

Most *HAC1* orthologs contain an unconventional intron that is excised to produce an active transcription factor (12, 40, 41). These introns are shorter in metazoan species (20–26 bases) compared with fungal species (>100 bases) except in some species in the fungal CTG clade (19–22 bases) (42, 43). Despite these differences in their lengths, sequences of both exon and intron junctions are shown to form a consensus stem–loop structure consisting of 7 bp stem and 7 bases loop containing an Ire1-cleavage motifs C xG∗CxGx (∗ = cleavage site and x = any nucleotides) (42, 44). Introns are cleaved by atypical splicing process during ER stress, resulting in production of an active transcription factor.

Introns of *HAC1* mRNA in *Saccharomyces* clade are long (252 bases in *S. cerevisiae*) (42) and uniquely associated with its 5′-UTR (Fig. 1*A*). This unique intron-5′-UTR association has a unique regulatory role in Hac1 protein expression at the translation level (6, 8). This class of *HAC1* mRNAs with long intron is regulated not only by atypical splicing during ER stress but also coordinated recruitment of translational components along with splicing. Consistent with the aforementioned notion, we found that expression of Hac1 protein from the unspliced *HAC1*-C(-27)G mRNA was enhanced when cells were grown in the presence of DTT (Fig. 1*C*). Together, these data imply that translational derepression in *HAC1* mRNA in *Saccharomyces* clade likely occur independent of cytosolic splicing. The question remains what would be of benefit to having two independent programs in *HAC1* mRNA translation in *Saccharomyces* clade. Identification of translational repressor(s), if any, might help understand the regulatory events in splicing and translational derepression.

Given that *HAC1* mRNA is associated with the helicase eIF4A (Fig. 8*C*), we propose a model (Fig. 8*F*) that the heterotrimeric eIF4F complex will form on the m^7^G cap and remain intact with the translationally silent *HAC1* pre-mRNA. We also propose that 30 RNA bases in between the 5′-cap and the UI RNA duplex in *HAC1* mRNA may not provide a sufficient space to recruit translational initiation factors, ribosomes, and Met-tRNA_i_^Met^. There are 23% of mRNAs in the budding yeast containing a 5′-UTR of ≤30 nucleotides (18), and many of those mRNA make proteins normally (www.yeastgenome.org). Therefore, it is likely that the UI RNA duplex prevents the dynamic recruitment of translation factors and inhibits the 43S PIC formation, rendering mRNA translationally inert. Thus, the *HAC1* mRNA translational system provides an excellent *in vivo* tool to study how a secondary structure near the 5′-cap modulates mRNA translation. Studies are underway to determine how the coordinated recruitment of translational factors facilitates translational repression in *HAC1* mRNA and how an alternate cap-dependent translation initiation stimulates protein synthesis from *HAC1* 5′-UTR mutants.

## Experimental procedures

### Yeast strains and growth conditions

Standard yeast extract peptone dextrose (YEPD) and SC with 2% dextrose media were used to grow and analyze the yeast strains of *S*. *cerevisiae*. Strains were grown in liquid or solid medium overnight at 30 °C. For ER stress induction, yeast cells were grown in YEPD, SC medium, or SC without appropriate amino acids at 30 °C to an absorbance value of ∼0.5 to 0.6 at 600 nm. The ER-stressor DTT (5 mM) or tunicamycin (0.5 μg/ml) was added to the medium, and cells were grown further for an additional 1 h (unless otherwise indicated). In general, we prefer to use tunicamycin for growth assays on the solid medium because it is more stable than DTT. All yeast strains used in this study are listed in Table 1.

**Table 1:** Yeast strains used in this study

Yeast strain	Genotype	Reference
WT (By4741)	*MATa his3-Δ1 leu2-Δ0 met5-Δ0 ura3-Δ0*	Deletion collection
*ire1Δ*	*MATa his3-Δ1 leu2-Δ0 met5-Δ0 ura3-Δ0 ire1*::*kanMX*	Deletion collection
*hac1Δ*	*MATa his3-Δ1 leu2-Δ0 met5-Δ0 ura3-Δ0 hac1*::*kanMX*	Deletion collection
*ire1Δ hac1Δ*	*MATa his3-Δ1 leu2-Δ0 met5-Δ0 ura3-Δ0 hac1*::*kanMX ire1*::*NatMX*	Lee *et al.* (44)

Plasmids were created using the standard gene manipulation techniques. Mutations were generated by appropriate DNA oligonucleotides, using the standard regular PCR or fusion PCR protocols. The desired mutation in each plasmid was confirmed by Sanger sequencing. All plasmids used in this study are shown in Table 2.

**Table 2:** Plasmids used in this study

Plasmid name	Plasmid	Reference
D3	pRS315, low-copy-number. *LEU2* vector	Lab collection
D4	pRS316, low-copy-number. *URA3* vector	Lab collection
D63	*HAC1* in D4	Sathe *et al.* (8)
D1243	HA-tagged *HAC1* in D4	This study
D1310	HAC1-C(-23)G in D4	This study
D1278	HAC1-C(-27)G in D4	Sathe *et al.* (8)
D2367	HAC1-C(-29)A in D4	This study
D2368	HAC1-C(-30)A in D4	This study
D2369	HAC1-C(-32)A in D4	This study
D2370	HAC1-C(-33)A in D4	This study
D2387	HAC1-A(-22)T, C(-23)G in D4	This study
D1822	HAC1^11bp^ in D4	This study
D2388	HAC1^9bp^ in D4	This study
D1775	HAC1^s^ in D4	This study
D2323	UI-HAC1^s^ in D4	This study
D1434	HAC1^12b^ in D4	This study
D1436	HAC1^24b^ in D4	This study
D1435	HAC1^36b^ in D4	This study
D1118	HAC1-AUG^-42^,A1G in D4	Sathe *et al.* (8)
D1378	HAC1-AUG^-42^,A1G^24b^ in D4	This study
D1041	eIF1 in a high-copy LEU2 vector	Hinnebusch (16)
D1043	eIF1A in a high-copy LEU2 vector	Hinnebusch (16)
D1195	eIF2α in a high-copy LEU2 vector	Hinnebusch (16)
D1193	eIF3a in a high-copy LEU2 vector	Hinnebusch (16)
D1045	eIF4A in a high-copy LEU2 vector	Hinnebusch (16)
D1206	eIF4B in a high-copy LEU2 vector	Hinnebusch (16)
D1197	eIF4E in a high-copy LEU2 vector	Hinnebusch (16)
D1201	eIF5 in a high-copy LEU2 vector	Hinnebusch (16)
D2122	eIF5B in a high-copy LEU2 vector	Hinnebusch (16)
D1205	Ded1 in a high-copy LEU2 vector	Hinnebusch (16)
D1420	FLAG-eIF4A in D3	This study
D1419	eIF4A in D3	This study

### WCE preparation and WB analysis

Cells were cultured under an ER stress condition as stated previously. WCEs were prepared by *trichloroacetic acid* method as described previously (45). Proteins in WCEs were fractioned by SDS–PAGE and subjected to WB analysis using antibodies against Hac1 (generated in our laboratory), phosphoglycerate kinase 1 (catalog no.: 459250; Invitrogen) or eIF2α (catalog no.: CM-217, gift from Thomas E. Dever, National Institutes of Health, USA). All experiments were repeated at least twice.

### Mutagenesis screen

Following the manufacturer's protocol, 50 μl chemically competent XL1-Red cells (catalog no.: 200129; Invitrogen) were transformed with the plasmid D1243 containing the hemagglutinin-tagged *HAC1* gene and plated on the LB medium containing ampicillin for 24 h. Cells were scrapped from the LB plate, and the mutator plasmids were isolated. A *hac1Δire1Δ* strain was transformed with the mutated D1243 plasmids, plated on YEPD medium overnight, and then replica printed on SC-uracil medium containing tunicamycin. The mutated plasmids from 30 tunicamycin-resistant colonies were rescued from yeast cells and sequenced to identify mutations. Subsequently, single mutations were generated as required.

### RT–PCR

Cells were cultured under an ER stress condition as stated previously. Cells were harvested, and total RNA was isolated using the RNeasy Mini Kit (Qiagen). Purified RNA was quantified using a Nanodrop spectrophotometer (ND-1000; Thermo Fisher Scientific). Purified RNA was used to synthesize the first-strand cDNA by a Superscript-III reverse transcriptase (catalog no.: 18080-093; Invitrogen) and a reverse primer (5ʹ-CCCACCAACAGCGAT AATAACGAG-3ʹ) that corresponded to nucleotides +1002 to 1025. To assay *HAC1* mRNA splicing, the synthetic cDNA was then PCR amplified using a forward primer (5ʹ-CGCAATCG AACTTGGCTATCC CTACC-3ʹ) that corresponded to nucleotides +35 to 60 and a reverse primer (5ʹ-CCCACCAACAGCGATAATA ACGAG-3ʹ) that corresponded to nucleotides +1002 to 1025. The PCR-amplified products were then run on a 1.5% agarose gel to separate spliced (HAC1^s^) and unspliced (HAC1^u^) forms of *HAC1* mRNA. Quantities of HAC1^s^ and HAC1^u^ were measured using ImageJ software (NIH). Experiments were repeated at least two times.

### Pulldown of eIF4A–RNA complex

The *ire1Δhac1Δ* strain coexpressing the untagged or FLAG-tagged eIF4A from a *LEU2* plasmid and the translationally repressed *HAC1* or translationally derepressed *HAC1*^*36nt*^ mRNA mutant from a *URA3* plasmid was grown in SC medium without uracil and leucine till the absorbance value reached ∼1.0 at 600 nm. Cells (absorbance at 600 nm = ∼50) were harvested and suspended in 1.2 ml of buffer A (20 mM Tris [pH 7.5], 50 mM KCl, and 10 mM MgCl_2_) supplemented with one EDTA-free protease inhibitor tablet per 10 ml buffer (Roche), 5 mM NaF, 1 mM DTT, 1 mM PMSF, and 1 μg/ml of the following protease inhibitors—pepstatin A, aprotinin, and leupeptin. Cells were broken with glass beads in a vortex mixer for 10 min at 4 °C and centrifuged at 10,000 rpm for 10 min in an Eppendorf 5810R refrigerated centrifuge. The supernatant was collected, further clarified by centrifugation at 13,000 rpm 10 min at 4 °C, and clear WCE was collected.

The amount of protein in WCE was measured by the standard Bradford protein assay. About 300 μg of protein was mixed with 30 μl of anti-FLAG-agarose resin (SIGMA) in buffer A containing 0.1% of Triton X-100. After 1 h, the tube containing the aforementioned mixture was centrifuged at 2500 rpm in an Eppendorf 5415R centrifuge for 1 min at 4 °C, and both supernatant (Sup) and pellet (P) fractions were collected. The pellet fraction was washed five times with the same buffer A, and the recovered protein was dissolved in the 2× SDS dye. Proteins in the supernatant fraction were precipitated with 20% trichloroacetic acid by standard protocol. Both pellet and supernatant fractions were subjected to WB analysis using an anti-FLAG antibody. About 10% of input of WCE was also used for WB analysis.

The amount of RNA in WCE was measured at an absorbance value at 600 nm using a spectrophotometer. About 300 μg of RNA was mixed with 100 μl of anti-FLAG-agarose resin (SIGMA) in buffer A containing 0.1% of Triton X-100. After 1 h, the tube containing the aforementioned mixture was centrifuged at 2500 rpm in an Eppendorf 5415R centrifuge for 1 min at 4 °C, and both supernatant (Sup) and pellet (P) fractions were collected. RNA was isolated from input, and supernatant and pellet fractions using the standard Trizol reagent (Invitrogen). Isolated RNA from the pellet fraction was subjected to DNase I treatment followed by RT–PCR analysis with *HAC1* mRNA-specific primers as described before.

### Bioinformatics analysis and RNA–RNA hybrid structure prediction

The transcriptome data were retrieved from the supporting information of an article published by Nagalakshmi *et al.* (18). We used the IntaRNA software (23), which predicts the RNA–RNA hybrids with duplex-binding structures by incorporating seed constraints and interaction site accessibility.

## Data availability

The authors confirm that the data supporting the findings of this study are available within the article.

## Conflict of interest

The authors declare that they have no conflicts of interest with the contents of this article.
